# Mitochondrial quality control protects photoreceptors against oxidative stress in the H_2_O_2_-induced models of retinal degeneration diseases

**DOI:** 10.1038/s41419-021-03660-5

**Published:** 2021-04-20

**Authors:** Biting Zhou, Lijun Fang, Yanli Dong, Juhua Yang, Xiaole Chen, Nanwen Zhang, Yihua Zhu, Tianwen Huang

**Affiliations:** 1 Department of Ophthalmology, The First Affiliated Hospital of Fujian Medical University, Fuzhou, Fujian China; 2 Department of Ophthalmology, Fujian Medical University Union Hospital, Fuzhou, Fujian China; 3 Qiqihaer Food and Drug Control Center, Qiqihaer, Heilongjiang China; 4 Department of Bioengineering and Biopharmaceutics, School of Pharmacy, Fujian Medical University, Fuzhou, Fujian China; 5 Department of Pharmacology, School of Pharmacy, Fujian Medical University, Fuzhou, Fujian China; 6 Department of Neurology, Fujian Medical University Union Hospital, Fuzhou, Fujian China; 7 Fujian Key Laboratory of Vascular Aging (Fujian Medical University), Fuzhou, Fujian China

**Keywords:** Cell death, Diseases

## Abstract

Retinal degeneration diseases (RDDs) are common and devastating eye diseases characterized by the degeneration of photoreceptors, which are highly associated with oxidative stress. Previous studies reported that mitochondrial dysfunction is associated with various neurodegenerative diseases. However, the role of mitochondrial proteostasis mainly regulated by mitophagy and mitochondrial unfolded protein response (mtUPR) in RDDs is unclear. We hypothesized that the mitochondrial proteostasis is neuroprotective against oxidative injury in RDDs. In this study, the data from our hydrogen peroxide (H_2_O_2_)-treated mouse retinal cone cell line (661w) model of RDDs showed that nicotinamide riboside (NR)-activated mitophagy increased the expression of LC3B II and PINK1, and promoted the co-localization of LC3 and mitochondria, as well as PINK1 and Parkin in the H_2_O_2_-treated 661w cells. However, the NR-induced mitophagy was remarkably reversed by chloroquine (CQ) and cyclosporine A (CsA), mitophagic inhibitors. In addition, doxycycline (DOX), an inducer of mtUPR, up-regulated the expression of HSP60 and CHOP, the key proteins of mtUPR. Activation of both mitophagy and mtUPR increased the cell viability and reduced the level of apoptosis and oxidative damage in the H_2_O_2_-treated 661w cells. Furthermore, both mitophagy and mtUPR played a protective effect on mitochondria by increasing mitochondrial membrane potential and maintaining mitochondrial mass. By contrast, the inhibition of mitophagy by CQ or CsA reversed the beneficial effect of mitophagy in the H_2_O_2_-treated 661w cells. Together, our study suggests that the mitophagy and mtUPR pathways may serve as new therapeutic targets to delay the progression of RDDs through enhancing mitochondrial proteostasis.

## Introduction

Retinal degeneration diseases (RDDs) such as age-related macular degeneration (AMD), retinitis pigmentosa (RP), and retinal detachment are multi-factorial diseases with a wide range of phenotypic and genotypic heterogeneity. They result in progressive photoreceptor degeneration and retinal dysfunction. Photoreceptor degeneration is the main pathologic basis of RDDs and the main cause of irreversible vision loss. Although the pathogenesis of RDDs has not been fully elucidated, it is clear that, as a metabolically active and high energy-demanding organ, exposure of the eye to environmental stimuli such as light, oxygen, and radiation plays a non-negligible role in the etiology of RDDs. Coupled with a high demand for oxygen, it makes photoreceptor cells extremely vulnerable to the insult of oxidative stress. Studies have revealed that retinal pigment epithelium (RPE) cells from AMD patients produced more reactive oxygen species (ROS) than cells in normal individuals^1,2^. Therefore, this suggests that oxidative stress-induced photoreceptor cell damage is likely involved in RDDs. In the early stage of RDDs, photoreceptor function is decreased, while the function of bipolar and ganglion cells remains normal. If photoreceptor function can be restored or the loss of photoreceptor cells can be reduced at this stage, remodeling of retinal neurons and glial cells and the formation of abnormal synapses will be impeded, thus improving the patient’s vision and restraining further development of the RDDs.

Mitochondria are the main power generators of the cell, and their function declines with aging. Studies have shown that mitochondrial dysfunction is associated with a variety of retinal diseases^3^. Mitochondrial quality control, including mitophagy and mitochondrial unfolded protein response (mtUPR), is important for maintaining mitochondrial proteostasis and cellular physiological functions. There are two types of mitophagy^4^. The first type is general autophagy, also known as a non-selective form of mitophagy; the second type is evident without autophagic vesicles and independent of mitochondrial fission. Mitochondrial damage caused by light radiation leads to the second type, in which the depolarized mitochondria are directly fused with structures containing microtubule-associated protein 1 light chain 3 (LC3) after being assembled with PINK1 and Parkin^4,5^. The impaired mitochondria release harmful molecules, including ROS and mitochondrial DNA (mtDNA) fragments, and activate the senescence-associated secretory phenotype (SASP)^6–8^. mtUPR involves the upregulation of HSP60, HSP10, CHOP, and other chaperones and proteases in mitochondria under stress conditions, promoting the degradation or correct folding of unfolded proteins in mitochondria. Unfolded proteins in the cytoplasm are imported into the mitochondria and degraded by the mtUPR, through a process called MAGIC (mitochondria as guardian in cytosol)^9^. When mitochondrial function is perturbed, mtUPR is activated to promote compartment fusion^10^ and mtDNA replication^11^ by retrograde signaling to the nucleus.

Lines of evidence have demonstrated that mitochondrial quality control has important protective effects in tumors, metabolic diseases, cardiovascular diseases, and neurodegenerative diseases. For example, mitophagy and mtUPR delay the progression of Alzheimer’s disease (AD) by enhancing mitochondrial proteostasis and reducing the deposition of Aβ proteins in the brain^12^. Additionally, both mitophagy and mtUPR can protect against pathogen infection by activating the innate immune response^13^. However, whether mitochondrial quality control has a neuroprotective role in oxidative stress-induced photoreceptor degradation has not been determined.

Therefore, the purpose of this study was to determine whether mitochondrial quality control has a protective effect against H_2_O_2_-induced oxidative damage of photoreceptors. To accomplish this, we examined functional and morphological alterations of 661w cells^14^, a mouse-derived cone cell line, under oxidative stress induced by H_2_O_2_.

## Materials and methods

### Cells culture and treatment paradigms

661w cells were purchased and validated from Aolu Biotechnology (Shanghai, China). The cells were cultured in high-glucose Dulbecco’s modified Eagle’s medium (DMEM) supplemented with 10% fetal bovine serum (FBS), 100 U/ml penicillin, and 100 μg/ml streptomycin. Cultures were maintained at 37 °C in a humidified atmosphere of 95% air and 5% CO_2_. After the cell confluence reached 80%, the cells were treated with 0–10 mmol/L hydrogen peroxide (H_2_O_2_; Amresco, OH, USA, Cat#E882). For the group treated with 1 mmol/L nicotinamide riboside (NR; Yuanye, Shanghai, China, Cat#S31692) or 10 μmol/L doxycycline (DOX; MedChem Express, NJ, USA, Cat#HY-N0565B) and H_2_O_2_, the cells were pretreated with NR or DOX for 24 h, then incubated with H_2_O_2_ for additional 12 h. For the group treated with 50 μmol/L chloroquine (CQ; MedChem Express, Cat#HY-17589) or 1 μmol/L cyclosporine (CsA; MedChem Express, Cat#HY-B0579), the cells were pretreated with CQ or CsA for 2 h before NR treatment. Incubation of 10 μg/mL oligomycin (MedChem Express, Cat#HY-N6782) and 1 μmol/L antimycin A (GLPBIO, CA, USA, Cat#GC42818) for 6 h was used as a positive control of mitophagy. NR, CsA, oligomycin, and antimycin A were dissolved in dimethysulfoxide (DMSO), while DOX and CQ were dissolved in distilled water. All the drugs were further diluted in the cell culture medium to achieve their working concentrations.

### Cell viability analysis

Cell viability was detected using 3-(4,5-dimethylthiazol-2-yl)-2,5-diphenyltetrazolium bromide (MTT; Yeasen, Shanghai, China, Cat#40201ES80). 661w cells were seeded in 96-well plates. After the indicated treatments, the cells were incubated in culture medium with 0.5 mg/ml MTT for 4 h. The formed dark blue crystals were dissolved with DMSO, and absorbance was measured at 570 nm using a microplate reader. Results are presented as the ratio of cell viability, taking the control as 1.

### Nuclear staining analysis by Hoechst 33342

The Hoechst 33342 staining solution was used to measure the change in cell survival. 661w cells were cultured and treated in 6-well plates. The 661w cells were then washed with phosphate-buffered saline (PBS), and stained with Hoechst 33342 dye (0.01 mg/mL) for 15 min at room temperature in the dark. After washing three times with PBS, the Hoechst-stained nuclei were visualized using a fluorescence microscope and the number of nuclei was calculated by Image J.

### Mitochondrial mass assay

Mitochondrial mass was detected by Mito-Tracker Red CMXRos (Beyotime, Shanghai, China, Cat#C1049). After the indicated treatment, 661w cells were incubated in medium containing 200 nmol/L Mito-Tracker Red CMXRos for 30 min. The nuclei were then stained with Hoechst 33342 dye for an additional 15 min. After washing with PBS, the samples were examined under a fluorescence microscope.

### Immunocytochemistry assay

661w cells were grown on confocal plates and fixed and permeabilized with methanol for 15 min at −20 °C, followed by three washes with PBS. Then the cells were incubated for 1 h in blocking buffer (5% normal goat or donkey serum, 0.3% Triton X-100 in PBS), following by incubation with anti-LC3B primary antibody (1:400, Cell Signaling Technology, MA, USA, Cat#83506), anti-TOM20 primary antibody (1:200, Cell Signaling Technology, Cat#42406), anti-PINK1 primary antibody (1:200, Abcam, Cambridge, UK, Cat#ab23707) and anti-Parkin primary antibody (1:200, Abcam, Cat#ab77924) overnight at 4 °C. After gently washing, the respective fluorescent secondary antibodies purchased from Abcam (Goat Anti-Mouse IgG H&L, Alexa Fluor 488, Cat#ab150113, 1:1000; Goat Anti-Rabbit IgG H&L, Alexa Fluor 647, Cat#ab150079, 1:1000; Donkey Anti-Mouse IgG H&L, Alexa Fluor 594, Cat#ab150108, 1:1000; Donkey Anti-Rabbit IgG H&L, Alexa Fluor 488, Cat#ab150073, 1:1000) were added and the cells were incubated at 37 °C for 2 h in the dark. Nuclei were stained with 0.01 mg/ml Hoechst 33342. The cells were photographed using a laser-scanning confocal microscope (Leica, Nussloch, Germany) for fluorescence and a digital camera driven by LAS AF software.

### Measurement of reactive oxygen species (ROS)

ROS was detected using a ROS assay kit (Solarbio, Beijing, China, Cat#CA1410). 661w cells were cultured and treated in 12-well plates, and then stained with DCFH-DA at 37 °C for 30 min. After three washes with PBS, the intensity of the fluorescence was measured using a fluorescence microscope.

### Detection of oxidative markers (SOD, CAT, and total GSH)

We used Total Superoxide Dismutase Assay Kit with WST-8 (Beyotime, Cat#S0101S) to measure the activity of SOD according to the manufacturer’s introductions. CAT activity was evaluated by commercially available CAT Assay Kit (Solaribo, Cat#BC0205) according to the introduction of manufacturer. The level of total GSH was measured using GSH and GSSG Assay Kit (Beyotime, Cat#S0053) according to the manufacturer’s protocol.

### Mitochondrial membrane potential (MMP) analysis

Following incubation and treatment of cells on 12-well plates, 661w cells were loaded with JC-1 dye (Solarbio, Cat#M8650) for 25 min. Next, the cells were washed three times with PBS and photographed immediately under a fluorescence microscope. For each well, JC-1 aggregates fluorescence (red) and monomer JC-1 fluorescence (green) were detected at the excitation/emission spectra of 560/595 nm and 485/535 nm, respectively. The red/green ratio was calculated as an indicator of MMP.

### Western blot

661w cells were cultured in 100 mm plates. After NR or DOX pretreatment and H_2_O_2_ incubation for the appropriate time, whole-cell extracts were obtained and resolved by denaturing sodium dodecyl sulfate-polyacrylamide gel electrophoresis (SDS-PAGE). Briefly, the collected cells were washed twice with ice-cold PBS, followed by resuspension in cell lysis buffer RIPA (Beyotime, P0013B) containing proteinase inhibitor PMSF (Beyotime, ST505, 1:100) and phosphatase inhibitor PhosSTOP (Roche, Mannheim, Germany, REF 04906845001). After incubation on ice for 30 min, debris was removed by centrifugation (12,000 *g*) at 4 °C and protein concentration was quantified by BCA assay, taking bovine serum albumin as a control. Proteins were diluted in ×5 loading buffer and denatured at 98 °C for 5 min. For western blot analysis, 20–40 μg proteins were separated by a 10% or 12% SDS-PAGE, and transferred onto a polyvinylidene fluoride (PVDF) membrane. The membrane was then incubated with primary antibodies against PINK1 (Abcam, Cat#ab23707), Parkin (Abcam, Cat#ab77924), LC3B (Cell Signaling Technology, Cat#83506), p62 (Abcam, Cat#ab77924), TOM20 (Cell Signaling Technology, Cat#42406), HSP60 (Cell Signaling Technology, Cat#12165), CHOP (Bioss, Beijing, China, Cat#bs20669R), Bcl-2 (Abcam, Cat#ab32124), β-actin (Abcam, Cat#ab8227) or β-Tubulin (Cell Signaling Technology, Cat#2146 S) at 4 °C overnight. All primary antibodies were diluted in a ratio of 1:1000. After washing with PBS, the membrane was incubated with the corresponding horseradish peroxidase (HRP)-conjugated goat anti-mouse (1:5000, Affinity Biosciences, OH, USA, Cat#S0002) or rabbit (1:5000; Affinity Biosciences, Cat#S0001) secondary antibody at room temperature for 2 h. Enhanced chemiluminescence was used to visualize the protein bands. All experiments were done at least three independent times, and Image J was used to analyze the gray value of each protein band.

### Detection of mitophagy

Lentiviral plasmid containing LC3-GFP was purchased from Hanbio Biotechnology (Shanghai, China). 661w cells were stably transfected with the lentiviral plasmid (MOI: 150), and then seeded onto confocal plates. After appropriate treatments, Mito-Tracker Red CMXRos was used to stain the mitochondria. Nuclei were stained using Hoechst 33342 dye. The fluorescent images were captured by laser-scanning confocal microscopy. Image J was used to analyze the co-localization of LC3 puncta and mitochondria.

### Statistical analysis

All experiments described were performed at least 3 times. Data presented as mean. Statistical analysis was performed using GraphPad Prism 7.0 (San Diego, CA, USA). Differences between the two groups were analyzed using Student’s t-test, whereas those among three or more groups were analyzed using one-way analysis of variance (ANOVA) with least significant difference test, accepting a significance level of *p* < 0.05.

## Results

### H_2_O_2_ induced oxidative damage of 661w cells

**Fig. 1:**
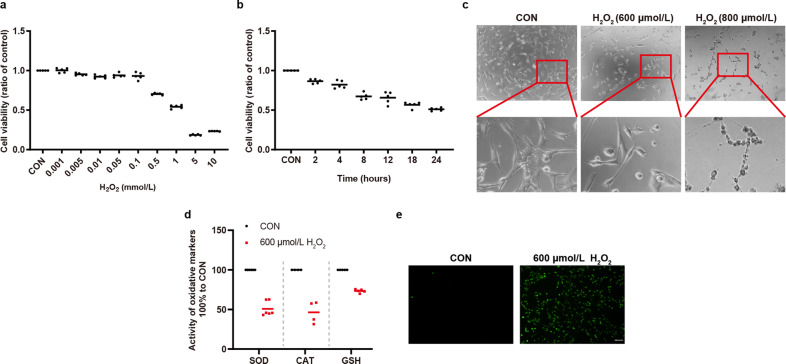
H_2_O_2_ induced oxidative damage to 661w cells. **a**, **b** H_2_O_2_ decreased cell viability in a dose- and time-dependent manner. 661w cells were treated with different concentrations of H_2_O_2_ for 12 h in (**a**) (*n* = 5). 600 μmol/L H_2_O_2_ was used to treat 661w cells for different hours in (**b**) (*n* = 5). **c** 661w cells became round and shrunken with 600 μmol/L H_2_O_2_ treatment. The morphology changes were irreversible with 800 μmol/L H_2_O_2_ treatment. **d** H_2_O_2_ inhibited the activity of SOD and CAT, and decreased the level of GSH (*n* ≥ 4). **e** H_2_O_2_ increased cellular ROS generation. Data are shown as mean. Scale bar: 100 μm.

Given that H_2_O_2_-induced oxidative stress can lead to a dose-dependent decrease of cell viability in various other cell types^15,16^, we sought to verify whether H_2_O_2_ can induce the death of photoreceptor cells in a dose-dependent manner. To this end, we determined the optimal concentration of H_2_O_2_ that could be used to establish an in vitro model of RDDs using the mouse retinal cone 661w cell line. As shown in Fig. 1a, there was a decrease in cell viability after 661w cells were exposed to 500 μmol/L H_2_O_2_ for 12 h. With an increase in H_2_O_2_ concentration and incubation time, the viability of 661w cells was decreased (Fig. 1a, b). As shown in Fig. 1c, 661w cells underwent morphological changes and became rounded and shrunken after treatment with 600 μmol/L H_2_O_2_. However, once the concentration of H_2_O_2_ was increased to 800 μmol/L, irreversible damage to 661w cells occurred. To further determine whether H_2_O_2_ can induce oxidative stress in our study, we measured the activity or level of anti-oxidant enzymes or substances including SOD, CAT, GSH, and ROS. The results showed that 600 μmol/L H_2_O_2_ inhibited the activity of SOD and CAT, and reduced the level of GSH, as well as promoted the generation of cellular ROS (Fig. 1d, e). Thus, the treatment with 600 μmol/L H_2_O_2_ was employed for subsequent experiments.

### NR-induced mitophagy in H_2_O_2_-treated 661w cells

**Fig. 2:**
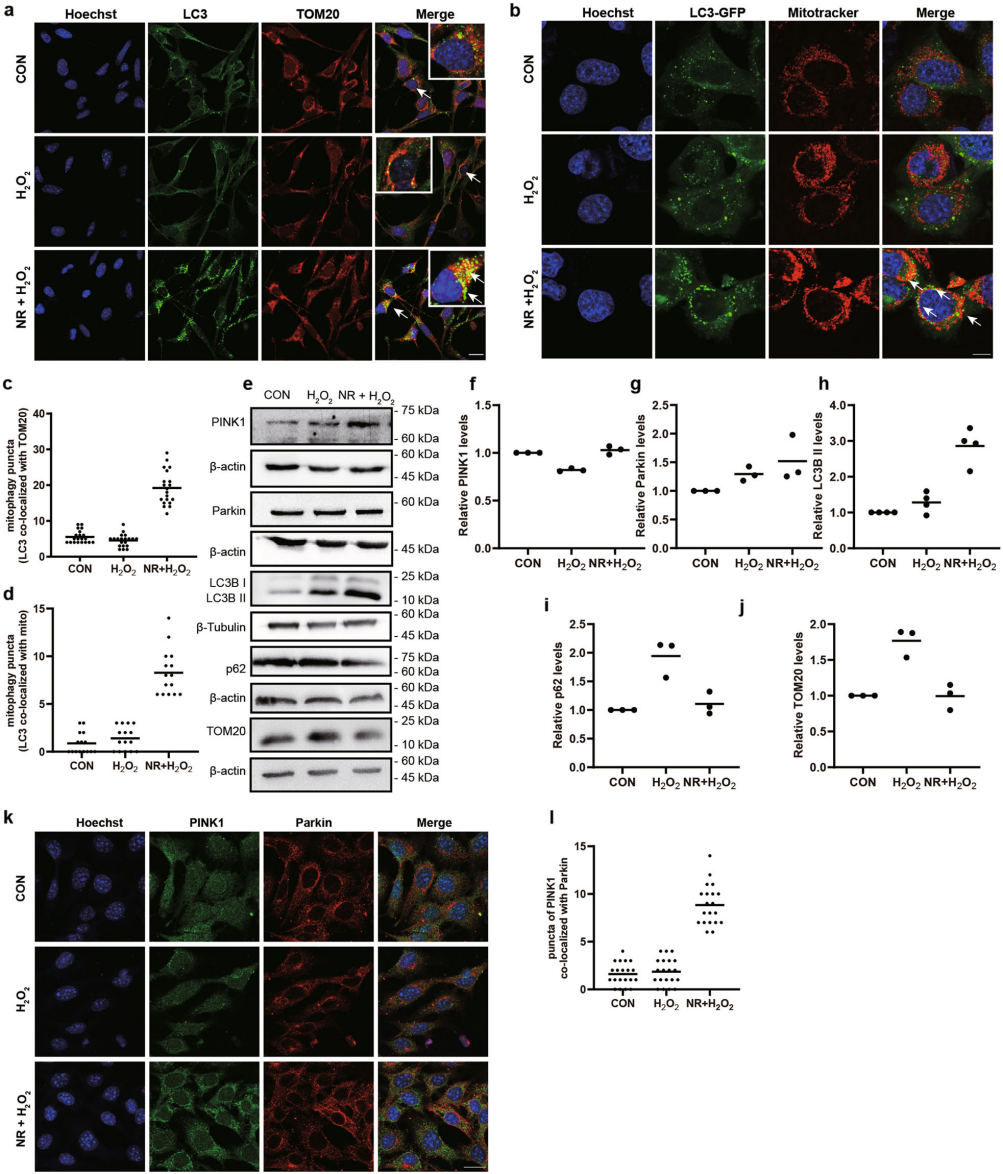
NR-induced mitophagy in H_2_O_2_-treated 661w cells. **a**, **c** Representative images of fluorescence-labeled mitophagy marker and mitochondrial marker. LC3 (green) and TOM20 (red) were immunolabeled, and nuclei were stained with Hoechst 33342 (blue). Scale bars: 25 μm. The number of yellow puncta (co-localization of LC3 and TOM20) were calculated in 20 cells and analyzed, showing activation of mitophagy in H_2_O_2_-treated 661w cells after NR pretreatment. **b**, **d** Representative images of 661w cells transduced with LC3-GFP virus and stained with Mito-Tracker Red CMXRos. LC3-GFP: green, mitochondrion: red and nuclei: blue. Scale bar: 10 μm. The number of mitophagy puncta were calculated in 15 cells and analyzed, showing NR increased the co-localization of LC3-GFP and mitochondria. **e**–**j** Western blot analysis of mitophagy-related proteins, PINK1, Parkin, LC3, p62, and TOM20. The relative expression of each protein was normalized to loading control (*n* ≥ 3). **k**, **l** Representative images of fluorescence-labeled PINK1 and Parkin. PINK1 (green) and Parkin (red) were immunolabeled, and nuclei were stained with Hoechst 33342 (blue). Scale bars: 25 μm. The co-localized puncta of PINK1 and Parkin were calculated in 20 cells and analyzed, showing the translocation of Parkin after NR treatment. Data are shown as mean.

It has been reported that NR activates mitophagy in various cell types^17,18^. To verify whether NR can induce mitophagy in 661w cells, we treated the cells with NR and used immunofluorescence and immunoblotting to detect the level of mitophagy. We found that the co-localized puncta of LC3 and TOM20 significantly increased in 661w cells pretreated with NR compared with that in the control and H_2_O_2_ groups (Fig. 2a, c). These results were confirmed in the 661w cells that were stably infected with a lentivirus containing an LC3-GFP construct and then treated with NR (Fig. 2b, d). It showed that the increased expression of the core proteins of mitophagy (LC3B II and PINK1), and the decreased expression of p62 and TOM20 (Fig. 2e–j). It’s worth noting that the increase of co-localization of Parkin with PINK1 in the NR-treated group, compared to that in the H_2_O_2_ group, was irrelevant to the level of Parkin (Fig. 2k, l). In agreement with previous studies^19–21^ and similar to NR, oligomycin, and antimycin A (O/A), a mitophagy activator, also induced mitophagy in our 661w cell model treated with H_2_O_2_ on the basis of the immunoblotting results from all examined makers but LC3B II (Fig. S1a–f). Taken together, all changes showed that mitophagy was activated by NR in 661w cells.

### Activation of mitophagy ameliorated mitochondrial injury and apoptosis of 661w cells under oxidative stress

**Fig. 3:**
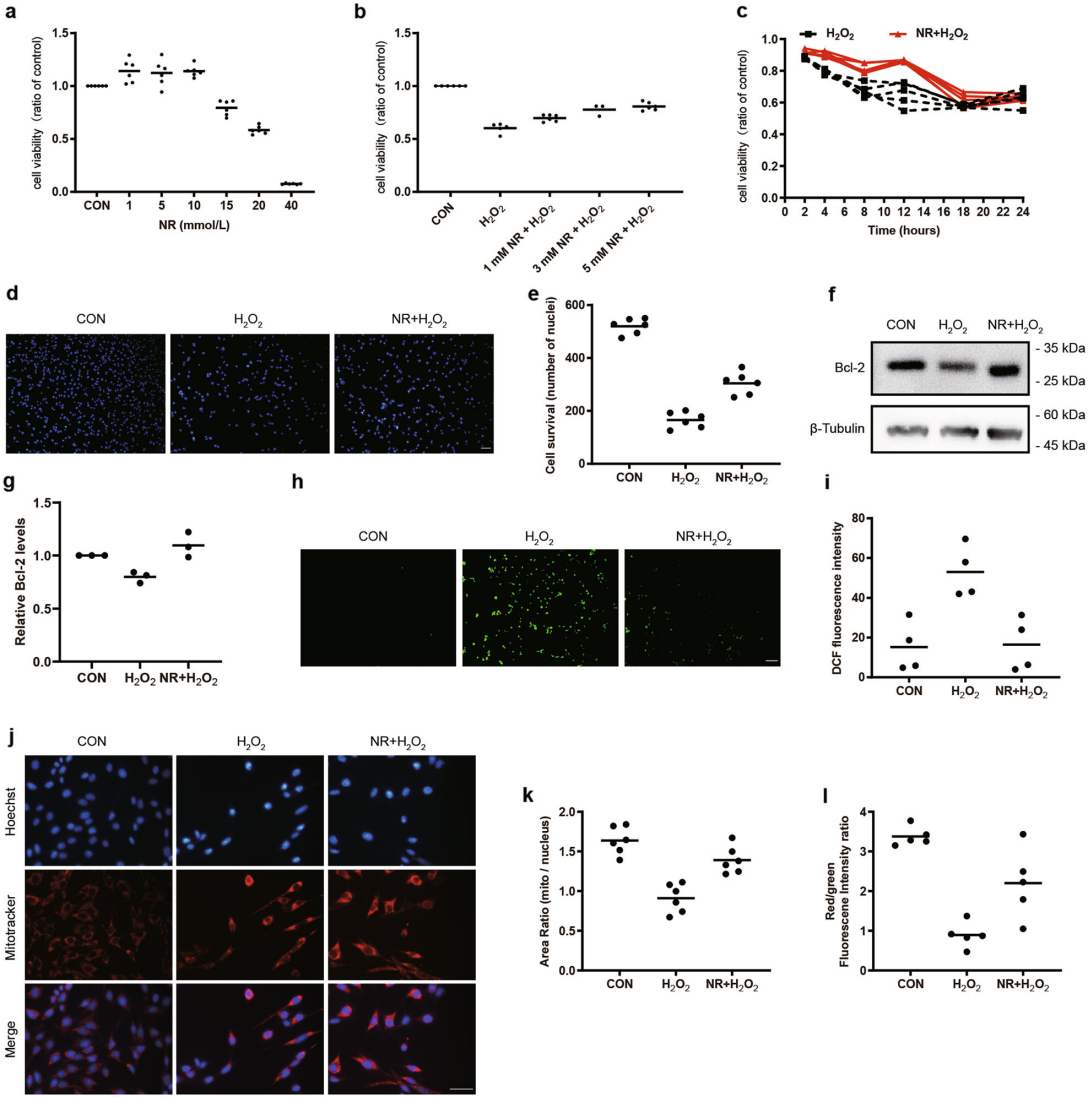
Protective effects of NR on H_2_O_2_-induced cellular damage of 661w cells. **a**–**c** Cell viability was determined by MTT assay. **a** NR increased cell viability of 661w cells (*n* = 6). **b** Attenuation of H_2_O_2_-induced decrease of cell viability after NR pretreatment (*n* ≥ 3). **c** The effect of 1 mmol/L NR pretreatment on 661w cells incubated with 600 μmol/L H_2_O_2_ for 2–24 h (*n* = 5). **d**, **e** Staining of nuclei by Hoechst 33342 showed cell survival (*n* = 6). **f**, **g** Immunoblotting analysis of Bcl-2 in 661w cells. The relative expression of Bcl-2 was normalized to the loading control (*n* = 3). **h**, **i** Inhibition of 600 μmol/L H_2_O_2_-induced ROS generation after 1 mmol/L NR pretreatment (*n* = 4). **j** Representative images of staining of Mitotracker (red) of 661w cells showed the mitochondrial mass, with the nuclei counterstained with Hoechst 33342 (blue). **k** Mitochondrial mass was analyzed by Image J, the intensity ratio of mitochondria/nuclei was calculated and compared among control, H_2_O_2_ and NR + H_2_O_2_ groups. NR attenuated H_2_O_2_-induced impairment of mitochondrial mass (*n* = 6). **l** 1 mmol/L NR pretreatment inhibited the 600 μmol/L H_2_O_2_-induced decline of mitochondrial membrane potential (*n* = 5). Data are shown as mean. Scale bar: 100 μm.

As shown by Fig. 3a, NR did not decrease, even slightly improved the cell viability of 661w cells within a certain concentration of 10 mmol/L. To further investigate the effect of mitophagy on the cellular viability of H_2_O_2_-treated 661w cells, the MTT assay and Hoechst 33342 staining were applied. The results showed that cell viability was higher in the NR + H_2_O_2_ group, when compared with the H_2_O_2_ group (Fig. 3b, d, e). In addition, when exposed to NR at the concentration of 1 mmol/L, pretreatment of the cells for 24 h prior to H_2_O_2_ treatment showed that the protective effects of NR peaked at 12 h after H_2_O_2_ treatment (Fig. 3c).

At the point of 12 h treatment, the cells were stained by Hoechst 33342. It showed that the NR ameliorated the death of 661w cells, which was the same with Fig. 3c. It further confirmed that the NR had protective function by activation of mitophagy during the damaged process induced by H_2_O_2_ in 661w cell.

To date, apoptosis is one important type of death, and the decrease of Bcl-2 expression and decrease of mitochondrial membrane potential (MMP) are the main changes for apoptosis. In our study, we found expression of Bcl-2 increased in the NR + H_2_O_2_ group, compared with the H_2_O_2_ group (Fig. 3f, g). MMP is an important parameter of apoptosis and mitochondrial function, which can be measured by a potential-dependent fluorescence shift from red to green with JC-1 staining. As shown by the red/green fluorescence intensity ratio in Fig. 3l, the proportion of 661w cells with high MMP decreased in the H_2_O_2_-treated group in comparison with the control 661w cells, suggesting an elevation of apoptosis and a decline of mitochondrial function. However, pretreatment with NR restored mitochondrial function and reduced apoptosis in H_2_O_2_-treated 661w cells (Fig. 3l).

Mitochondria are a major producer of ROS, and ROS generation is used as another indicator of mitochondrial function. The level of intracellular ROS was measured using a DCFH-DA probe. As shown in Fig. 3h, i, H_2_O_2_ markedly increased the ROS production (green fluorescence) compared with the control group, while the NR + H_2_O_2_ group showed an attenuation of cellular ROS generation. We further explored the effect of mitophagy on mitochondrial biogenesis through staining with Mito-Tracker, a specific mitochondrial dye and a marker for assessing mitochondrial mass, which revealed that the mitochondrial mass in the H_2_O_2_-treated group was lower than that in both the control and the NR + H_2_O_2_-treated groups (Fig. 3j, k). Interestingly, we found that pretreatment H_2_O_2_-induced 661w cells with O/A did not impede cell apoptosis (Fig. S1g, h) and loss of mitochondrial mass (Fig S1i, j).

### Inhibition of mitophagy reversed the protective effects of NR on H_2_O_2_-treated 661w cells

**Fig. 4:**
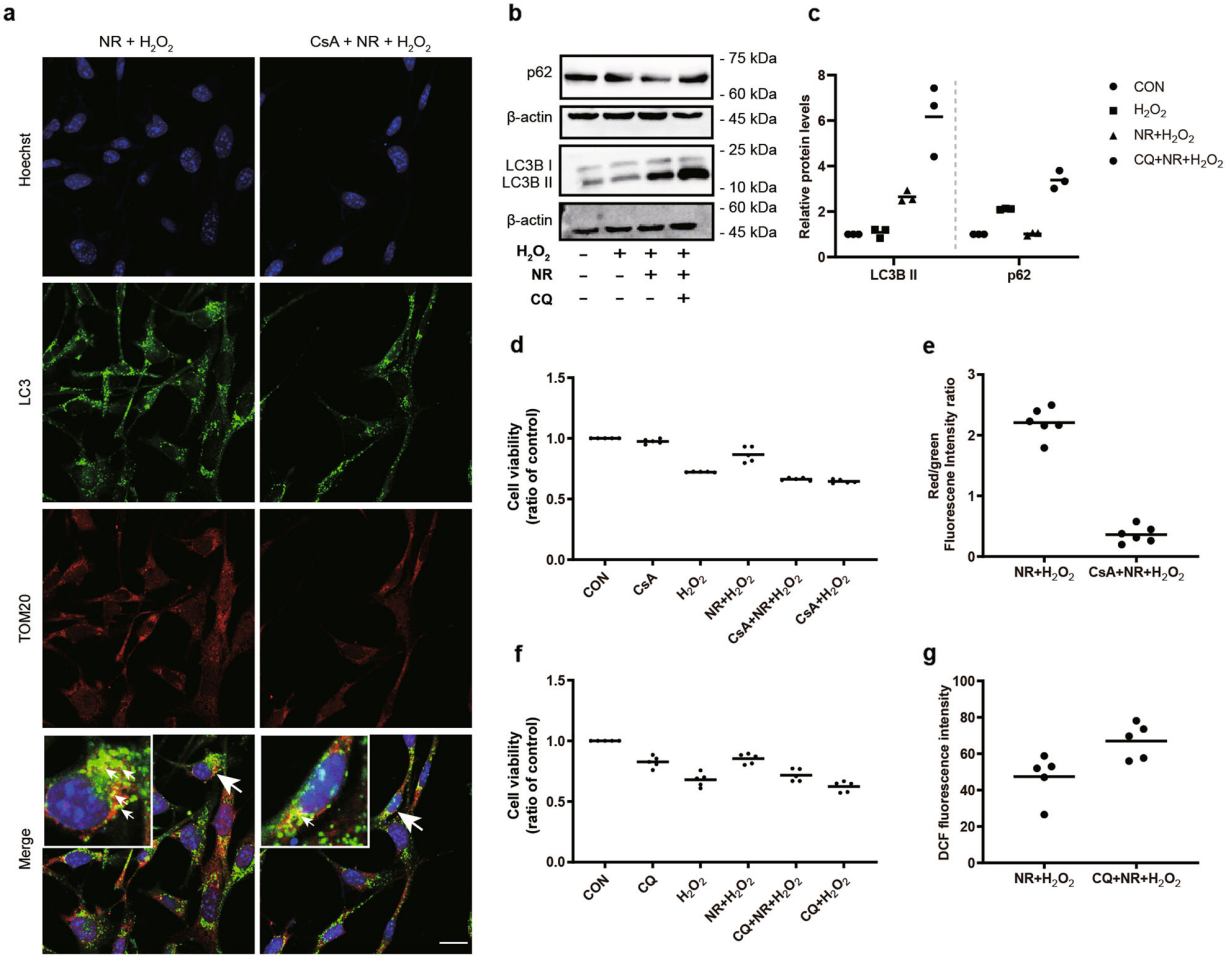
Inhibition of mitophagy decreased the protective effects of NR on H_2_O_2_-treated 661w cells. **a** Inhibition of NR-induced increase of co-localized puncta (LC3 and TOM20) after 1 μmol/L CsA pretreatment. **b**, **c** Western blot analysis of LC3 and p62. The relative expression of LC3B II and p62 were normalized to β-actin (*n* ≥ 3). **d**, **f** Cell viability in the CQ + NR + H_2_O_2_ and CsA +NR + H_2_O_2_ treatments was lower than that of the NR + H_2_O_2_ group (n = 5). **e** 1 μmol/L CsA pretreatment inhibited the NR-induced increase of mitochondrial membrane potential in H_2_O_2_-treated 661w cells (*n* = 6). **g** 50 μmol/L CQ pretreatment inhibited the NR-induced decline of ROS generation in H_2_O_2_-treated 661w cells (*n* = 5). Data are shown as mean. Scale bar: 25 μm.

In order to further investigate the effect of mitophagy on regulating 661w cell function under oxidative stress, we used cyclosporin A (CsA) and chloroquine (CQ) as mitophagy inhibitors, according to previous studies^22–24^. CsA decreased the co-localized puncta of LC3 with TOM20 (Fig. 4a), and CQ increased the expression of LC3B II and p62 (Fig. 4b, c), which conformed that CsA and CQ are inhibitors of mitophagy. The increase in cell viability caused by NR pretreatment of H_2_O_2_-treated 661w cells was reversed by CsA and CQ pretreatment (Fig. 4d, f). As shown in Fig. 4e, a decrease of MMP was detected in the CsA+NR + H_2_O_2_ group compared with that in the NR + H_2_O_2_ group. In addition, the 661w cells treated with CQ + NR + H_2_O_2_ generated higher ROS than the cells in the NR + H_2_O_2_ group (Fig. 4g), suggesting that mitophagy is essential for 661w cell survival and mitochondrial function.

### DOX-induced mtUPR inhibited mitochondrial injury and apoptosis of 661w cells under oxidative stress

**Fig. 5:**
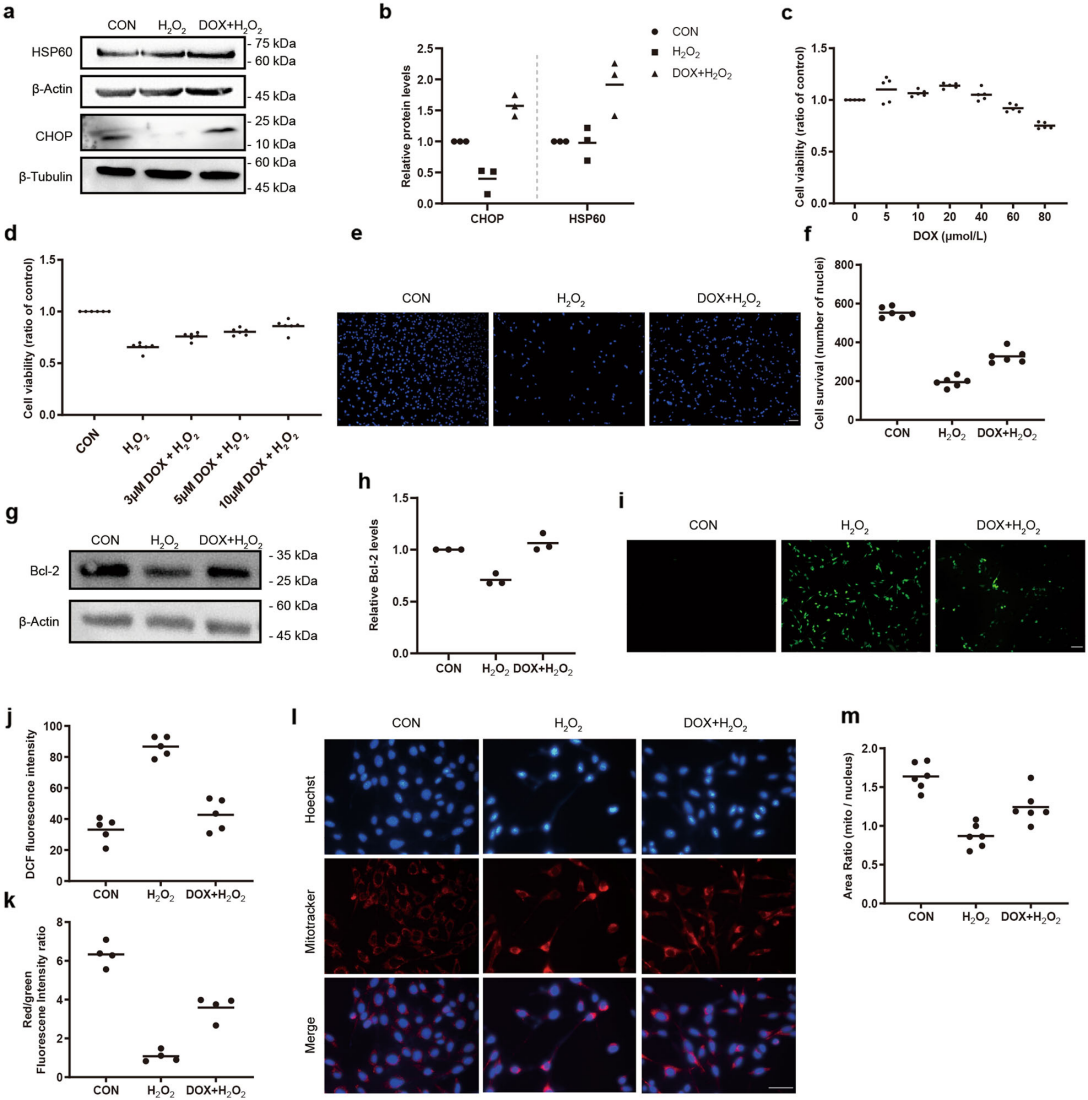
Protective effects of DOX-induced mtUPR on H_2_O_2_-induced cellular damage of 661w cells. **a**, **b** Western blot analysis of mtUPR-related proteins showed the upregulation of HSP60 and CHOP in H_2_O_2_-induced cells after 10 μmol/L DOX pretreatment. The relative expression of HSP60 and CHOP were normalized to β-actin and β-Tubulin, respectively (*n* = 3). **c**, **d** 10 μmol/L DOX increased cell viability in 661w cells with or without 600 μmol/L H_2_O_2_ stimulation with MTT assay (*n* ≥ 5). **e**, **f** The cell survival in the DOX + H_2_O_2_ group was higher than that of the H_2_O_2_ group based on Hoechst 33342 staining (*n* = 6). **g**, **h** 10 μmol/L DOX increased the expression of Bcl-2 in 600 μmol/L H_2_O_2_-treated 661w cells by western blotting. The relative expression of Bcl-2 was normalized to β-actin (*n* = 3). **i**, **j** Representative images of ROS and analysis of fluorescence intensity (*n* = 5). **k** Attenuation of 600 μmol/L H_2_O_2_-induced decrease of mitochondrial membrane potential after 10 μmol/L DOX pretreatment (*n* = 4). **l**, **m** Improvement of 600 μmol/L H_2_O_2_-induced reduction of mitochondrial mass after 10 μmol/L DOX pretreatment (*n* = 6). Data are shown as mean. Scale bar: 100 μm.

Because mitochondria are derived from bacteria, antibiotics such as DOX^25^ that inhibit bacterial translation can also be used to inhibit mitochondrial protein translation. Therefore, we used DOX to determine the role of the mtUPR in 661w cells under oxidative condition. DOX increased the expression of CHOP and HSP60, two core proteins of the mtUPR pathway (Fig. 5a, b). 20 μmol/L of DOX increased the cell viability, whereas 80 μmol/L of DOX acted oppositely on the cell viability (Fig. 5c). MTT assay (Fig. 5d) and Hoechst 33342 staining (Fig. 5e, f) revealed that DOX promoted cell survival when 661w cells were treated with H_2_O_2_. Moreover, increased expression of Bcl-2 was detected in the DOX + H_2_O_2_ group, compared with that in the H_2_O_2_ group (Fig. 5g, h). To further determine the effect of the mtUPR on mitochondrial function, we measured ROS fluorescence intensity, MMP, and mitochondrial mass. Under oxidative stress, H_2_O_2_ led to a profound increase of ROS, and a marked decrease of MMP and mitochondrial mass. However, the cells pretreated with DOX showed a reduction of ROS, and increases of MMP and mitochondrial mass, suggesting that the mtUPR is one of the important ways for maintaining mitochondrial homeostasis in 661w cells (Fig. 5i–m).

## Discussion

The eye is a complex organ that provides information on the form, the light intensity, and the color reflected from objects. Retinal degeneration is one of the most common forms of visual impairment in the world, which is caused by neural degeneration of photoreceptor cells, bipolar cells, and retinal ganglion cells. The photoreceptor cells, including rods and cones, are critical mediators for light transduction. Their death is the endpoint of many blinding diseases. In many retinal degenerative diseases, the impairment of photoreceptor cells occurs in the early stage. Thus, preventing the early pathological process is highly valuable for impeding retinal degeneration. Although the mechanism(s) of photoreceptor impairment and death are not fully elucidated, oxidative stress is a key factor during retinal degeneration. Hydrogen peroxide (H_2_O_2_), as one of the ROS inducers, has been widely used to induce cellular oxidative stress and cellular death. 661w cells are a mouse-derived retinal cone cell line. Although they lack structural outer segments, 661w cells have similar cytological and biological characteristics to cone cells^26,27^, and spindle-like processes that are characteristic of neuronal morphology^14^. Therefore, 661w cells are often used for in vitro studies of photoreceptors^28–30^.

In our paper, the retinal degeneration of 661w cells was induced by H_2_O_2_ in a time- and dose-dependent manner. Our data suggest that oxidative stress has a role in impairing the viability of 661w cells. Additionally, the increase of ROS and the decrease of mitochondrial transmembrane potential suggest that mitochondrial dysfunction is involved in the pathological processes. Upon mitochondrial defects, several types of quality control machinery are activated in mitochondria, including the mitochondrial-specific molecular chaperones and proteases for prevention of protein misfolding at the molecular level (mtUPR) and elimination of dysfunctional mitochondria at the organelle level (mitophagy). Previous studies^5,12,31,32^ have shown that mitophagy and the mtUPR play a role in the process of neurodegenerative diseases. However, the role of mitophagy and the mtUPR in retinal degenerative diseases remains unclear. In our paper, we demonstrated that the activation of mitophagy and the mtUPR enhance the photoreceptor’s ability to resist the damages caused by H_2_O_2_-induced oxidative stress, inhibit cellular apoptosis/death, and repair mitochondrial function. This suggests that mitophagy and the mtUPR are potential targets for the therapy of retinal degeneration.

It is well-known that in the PINK1/Parkin-mediated mitophagy pathway, mitochondrial depolarization stabilizes the PINK1 protein on the outer mitochondrial membrane, and recruits Parkin protein from the cytoplasm to the outer mitochondrial membrane, forming a ubiquitin chain, which is recognized by ubiquitin-binding protein p62. The conjugation of p62, ubiquitin, and LC3 induces the formation of mitochondrial autophagosomes.

In our study, we found that NR, an NAD^+^ precursor, as a mitophagic activator^17,18^, prompted the mitophagic response by up-regulating the expression of PINK1 and LC3B II, inducing the co-localization of PINK1 and Parkin, and reducing the level of p62. In addition, the imaging analysis of LC3-GFP confirmed that NR is able to activate mitophagy. By contrast, CQ, an autophagic inhibitor^33^, and CsA, a mitophagic inhibitor^22,23^ inhibited mitophagy. A combination of oligomycin and antimycin A was reported to initiate mitophagy by inhibiting mitochondrial respiration and impairing mitochondrial homeostasis. Although the use of O/A promotes the clearance of damaged mitochondria, the mitochondrial dysfunction caused by O/A actually induces cell apoptosis, which is completely different from NR in the 661w cell model induced by oxidative stress.

The mtUPR is a transcriptional response that is activated by various forms of mitochondrial dysfunction and is regulated by mitochondrial-nuclear communication^25,34^. The mitochondrial dysfunction induced by knockout of mtDNA causes an increase of mitochondrial chaperones (HSP60, HSP10) and matrix proteasomes (ClpP). In addition, mutation(s) of the mitochondrial matrix protein ornithine transcarbamylase (OTC) leads to activation of the mtUPR. CHOP is another important protein in response to the mtUPR. The transcriptional activation of CHOP by the mtUPR reflects the level of unfolded protein in the mitochondrial matrix, and is consistent with inhibition of the mtUPR by mutated CHOP^35^. Therefore, HSP60, HSP10, ClpP, and CHOP are the main proteins which reflect the activation of the mtUPR. It has been reported that DOX activates the mtUPR by inhibiting the translation of mitochondrial proteins^22^. In our study, we also found that the mtUPR activated by DOX induced the increased expression of HSP60 and CHOP, resulting in the attenuation of H_2_O_2_-induced loss of mitochondrial mass.

Of note, we showed that the activation of both mitophagy and the mtUPR can reduce ROS generation in photoreceptor cells and inhibit cell apoptosis, while mitophagy inhibitors reverse these protective effects. It is likely that mitophagy and the mtUPR maintain the balance between normal and damaged mitochondria by eliminating unnecessary mitochondria, unfolded proteins, ROS, cytochrome c and other apoptosis-related factors in photoreceptor cells, which ensures the energy supply and the biological functions of cells.

Under stress conditions, cells maintain mitochondrial protein homeostasis and mitonuclear protein balance through the mtUPR on the one hand, and clear damaged mitochondria through the autophagy-lysosomal pathway on the other hand, and use mitophagy products to regenerate normal mitochondria, together ensuring the physiological functions of mitochondria. It is worth noting that although the mtUPR can repair temporary mitochondrial dysfunction caused by toxins, pathogens^13,25,36^ or mutations in nuclear genes encoding the respiratory chain^37,38^, increasing the contents of deleterious mtDNA will disturb or delay the mtUPR, eventually leading to age-related diseases^39,40^. These data are in agreement with ours. Furthermore, the accumulation of unfolded protein in mitochondria activates the mtUPR, resulting in an increase in the production of chaperones to promote the correct folding of proteins or degradation of proteins by proteases. On the other hand, the depolarized mitochondria are cleared by mitochondrial autophagy through the PINK1/Parkin pathway. Taken together, these data suggest that the increase of mitophagy and the mtUPR have a protective role under stress.

The crosstalk of mitophagy and the mtUPR is important in mitochondrial quality control, but how both of them communicate is unclear. Although our study did not explore the link between mitophagy and the mtUPR in 661w cells under H_2_O_2_ stress, some scholars^41^ believe that the mtUPR is an early response to restore protein homeostasis under stress. When the damage exceeds the repair capacity of the mtUPR, mitophagy is activated to clear the damaged or aging mitochondria. One study found that the mitophagy receptor protein FUNDC1 located on the outer membrane of mitochondria can interact with the cytoplasmic chaperone protein HSC70, providing a new explanation for the interplay of mitophagy and the mtUPR^42^. Although the link between mitophagy and the mtUPR is complicated and needs to be further investigated, our data have confirmed that both of them exert protective effects on photoreceptors, providing a new insight into the clinical treatment of RDDs.

**Fig. 6:**
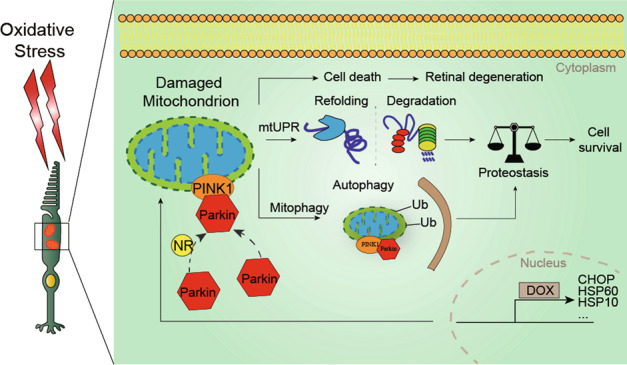
Schematic representation of the events that take place in 661W cells with H_2_O_2_. Upon H_2_O_2_ treatment, the mitochondria damaged by ROS overproduction and mitochondrial membrane potential collapse. This led to 661W cell death due to oxidative stress. However, at the same time, the mtUPR and PINK1-Parkin induced mitophagy are activated to respond to the mitochondrial damage, which had protective functions for cellular survival.

Taken together (Fig. 6), our findings demonstrate that mitophagy and the mtUPR play a vital role in 661w cells under oxidative stress, indicating that both of them can serve as therapeutic targets for photoreceptor degeneration caused by oxidative stress.

## Supplementary information

Supplementary information

## Data Availability

The datasets used during the current study are available from the corresponding author on reasonable request.
