# Supplementary figures and images for: Mitochondrial quality control protects photoreceptors against oxidative stress in the H2O2-induced models of retinal degeneration diseases
Source: Cell Death Dis. 2021 Apr 20;12(5):413. doi: 10.1038/s41419-021-03660-5 (PMC8058096; doi:10.1038/s41419-021-03660-5)

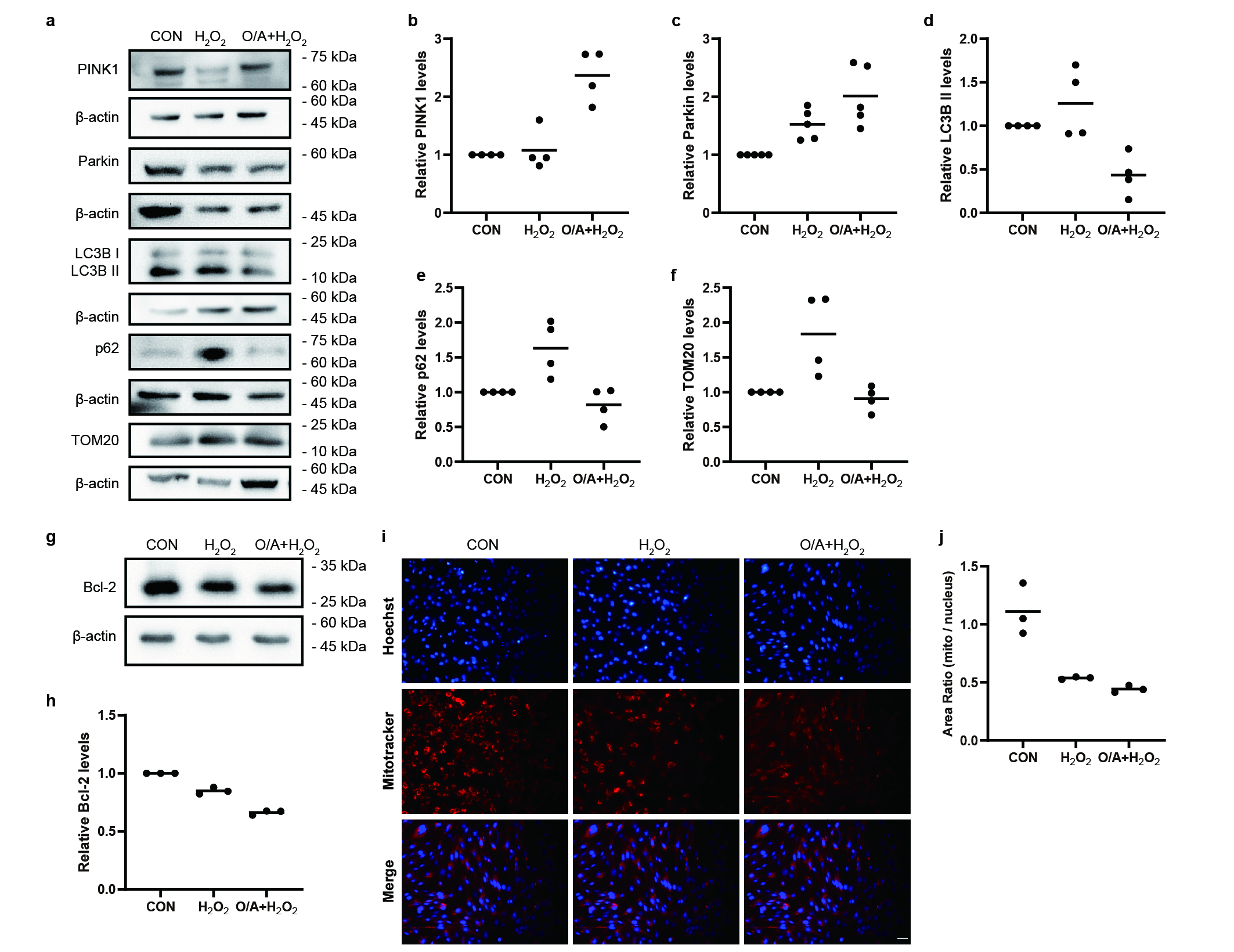

Supplement: Supplementary file 1 — Supplementary information [file 41419_2021_3660_MOESM1_ESM.tif]
